# A comparison of clinical outcomes between simultaneous integrated boost (SIB) versus sequential boost (SEQ) intensity modulated radiation therapy (IMRT) for head and neck cancer: A meta-analysis

**DOI:** 10.1097/MD.0000000000016942

**Published:** 2019-08-23

**Authors:** Li Jiang, Yong Zhang, Zhendong Yang, Feifei Liang, Jiangtao Wu, Rensheng Wang

**Affiliations:** Department of Radiation Oncology, The First Affiliated Hospital of Guangxi Medical University, Radiation Oncology Clinical Medical Research Center of Guangxi, Nanning, Guangxi, China.

**Keywords:** clinical outcomes, head and neck cancer, meta-analysis, SEQ-IMRT, SIB-IMRT

## Abstract

**Background:**

The optimal intensity modulated radiation therapy (IMRT) technique for head and neck cancer (HNC) has not been determined yet. The present study aimed to compare the clinical outcomes of the simultaneous integrated boost (SIB)-IMRT versus the sequential boost (SEQ)-IMRT in HNC.

**Methods:**

A meta-analysis of 7 studies involving a total of 1049 patients was carried out to compare the treatment outcomes together with severe acute adverse effects of the SIB-IMRT versus the SEQ-IMRT in HNC patients.

**Results:**

Comparison of the SIB-IMRT and SEQ-IMRT showed no significant difference in the measurement of overall survival (OS) (hazard ratio [HR] 0.94; 95% confidence inerval [CI], 0.70–1.27; *P* = .71), progression free survival (PFS) (HR 1.03; 95% CI, 0.82–1.30; *P* = .79), locoregional recurrence free survival (LRFS) (HR 0.98; 95% CI, 0.65–1.47; *P* = .91), and distance metastasis free survival (DMFS) (HR 0.87; 95% CI, 0.50–1.53; *P* = .63). Moreover, there were no significant differences in adverse effect occurrence between the SIB-IMRT and SEQ-IMRT groups.

**Conclusion:**

SIB-IMRT and SEQ-IMRT can provide comparable outcomes in the treatment of patients afflicted by HNC. Both IMRT techniques were found to carry a similar risk of severe acute adverse effect. SIB-IMRT may have advantages due to its convenience and short-course of treatment; however, the optimum fractionation and prescribed dose remained unclear. Furthermore, both IMRT techniques can be advocated as the technique of choice for HNC. Treatment plan should be individualized for patients.

## Abstract 2

Supplemental Digital Content is available in the text

## 1 Introduction

Radiation therapy for head and neck squamous cell carcinoma has become mainstream treatment over the past decades with the advent of intensity modulated radiation therapy (IMRT). The IMRT technique is characterized by a highly conformal dose distribution to targets, whereas a constraint dose to organs at risk (OARs).^[1]^ Historically, sequential boost (SEQ) intensity modulated radiation therapy regimens for head and neck cancer (HNC) are composed of an elective irradiation followed by a series of reduced boost fields aiming at the different overall doses needed for tumor control or OARs tolerance. In other words, the treatment planning of SEQ-IMRT was built on the experience obtained from the era of conventional radiotherapy.^[2]^ As a result, several treatment plans are being designed for each patient, with each risk area receiving the same dose per fraction. This dose usually ranges from 1.8 to 2 Gy to minimize radiation toxicity. In contrast, IMRT with simultaneous integrated boost technique allows the simultaneous delivery of individualized dose levels of selective target volumes (TV) by generating one single treatment plan. Simultaneous integrated boost (SIB) technique gained popularity as it improved planning efficiency and escalated the dose per fraction delivered to the gross target volume (GTV) to potentially enhance tumor control.^[3,4]^ Several studies have reported that SIB-IMRT is a safe and effective treatment for HNC, whereas it offers the following advantages: (1) shortening of the treatment time; (2) increased biologically equivalent dose (BED) to the tumor with dose per fraction slightly >2 Gy; and (3) more conformal dose distributions compared with that of SEQ-IMRT which is divided into a large-field phase and a boost phase.^[5–7]^ Nonetheless, some studies showed that SIB-IMRT might present a risk of locoregional failure due to the lower marginal doses. Therefore, patients undergoing SIB-IMRT were susceptible to side effects when the doses given to the adjacent critical structures or other normal tissues were the major concern in the high-dose region.^[8,9]^ In summary, there is still a paucity of powerful evidence with respect to the pros and cons of SIB-IMRT and SEQ-IMRT. In an effort to evaluate the advantages and disadvantages of the SIB and SEQ techniques, we aimed at obtaining more credible evidence in regard to these 2 techniques. That being the case, we performed a meta-analysis comparing the clinical outcomes of SIB-IMRT and SEQ-IMRT.

## 2 Materials and methods

### 2.1 Search strategies

The eligible studies were identified by systematically searching the electronic databases, including PubMed, Embase, The Cochrane Library, ClinicalTrials.gov, Biosis, Web of science, Chinese National Knowledge Infrastructure, Chinese Wanfang and Chongqing VIP up to the December 1, 2019, without any language restriction. Our search strategy was composed of the following terms: (1) “carcinoma” or “ cancer”; (2) “ IMRT”; (3) “pharyngeal” or “head and neck” or “oral” or “laryngeal” or “tongue” or “tonsil” or “nose” or “nasal” or “paranasal sinus” or “lip” or “cheek” or “palatal” or “cervical esophageal”; and (4) “randomized controlled trial ” or “retrospective study” or “ prospective study” or “trial” or “outcome.” The above terms were used for retrieval in the following combinations: (1) and (2) and (3) and (4).

### 2.2 Study selection

Titles and abstracts were screened and assessed according to the following eligibility criteria: (1) patients who were diagnosed with typical morphology and/or immunopathology based on the WHO classifications; (2) patients who were newly diagnosed without any second primary malignancy; (3) studies that were designed as randomized controlled trials were a priori included; if not, then other interventional studies that compared the outcomes of SIB-IMRT and SEQ-IMRT were included; (4) treatments which were performed with curative intent; (5) main measurement of the outcome to be the hazard ratio (HR) for overall survival (OS), progression-free survival (PFS), distant metastases-free survival (DMFS), local recurrence-free survival (LRFS); and odds ratio (OR) or risk ratio (RR) for side effect which could be extracted and calculated from the full-text article; (6) data such as 5-year OS and 5-year PFS could be extracted and calculated from the full-text article; and (7) when data overlapped between articles, we chose the most integrated study. Ethics approval and patient written informed consent were not required because all of the analyses in our study were performed based on data from already published studies.

### 2.3 Data extraction

Two independent authors carefully extracted data from all of the included studies, a process which was additionally checked by a third author. For each eligible study, the following information was extracted: first author, year of publication, patient characteristics and numbers, study design, survival curves based on the Kaplan–Meier method, comparison of outcomes with *P* values, events related to side effects, and systemic and locoregional failure.

### 2.4 Quality assessment

The methodological quality of each eligible study was evaluated independently by 2 investigators, using the Newcastle–Ottawa quality assessment scale for studies.^[10]^ Any disagreements between the 2 investigators were settled by a third one. The quality assessment score ranged from 0 to 9 points. Studies with a score of ≥7 were considered as high quality, which characterized them as exhibiting a good design and abundant information.

### 2.5 Statistical analyses

Review Manager (RevMan) 5.3 software was utilized for all of the statistical analyses. For time-to-event variables, the effect of each study was expressed as HR with a 95% confidence interval [CI], which was calculated according to the methods described by Tierney et al.^[11]^ For dichotomous variables, OR or RR with a 95% CI was used to describe the effect. The effect was meaningful when the *P* value was <0.05. Statistical heterogeneity of the effect was evaluated by the Cochrane *Q* test and *I*^2^. Subsequently, the result was analyzed by a fixed-effects model or random-effects model according to the heterogeneity: a fixed-effects model was applied when *I*^2^ < 50% and *P* > .1; otherwise a random-effects model was utilized.^[12]^ When heterogeneity was detected, sensitivity analysis was employed to detect its potential sources by investigating the effect of each omitted study on the overall estimate. Publication bias was estimated by the funnel plot using the RevMan software.

## 3 Results

### 3.1 Study selection

The selection process for the included studies can be found in the flow diagram (Supplementary material). A total of 2659 records were marked with the initial literature search. The preliminary screening depending on titles and abstracts identified 21 studies as potentially relevant, which were subsequently retrieved in full text review. Among these, 14 studies were excluded because they did not reach the inclusion criteria. After the application of the eligibility criteria, 7 studies involving a total of 1049 patients for pooled estimation were finally included in the analysis.^[13–19]^

### 3.2 Studies’ characteristics

Six of the 7 articles were published in English and the other one in Chinese. The year of publication ranged from 2014 to 2018. Based on the quality evaluation, all of the included studies were of high quality (score ≥7). Three out of 7 studies were designed as randomized controlled trials and the remaining ones followed the study design of a retrospective research. There was variability among the studies in terms of study design, sample size, methods, site of primary lesions, and treatment protocols. The core data, which presented the basic characteristics of patients, are shown in Table 1. According to the statistics of each study, the 2 groups did not differ significantly based on ethnic, comorbidities, stage or primary site.

**Table 1:**
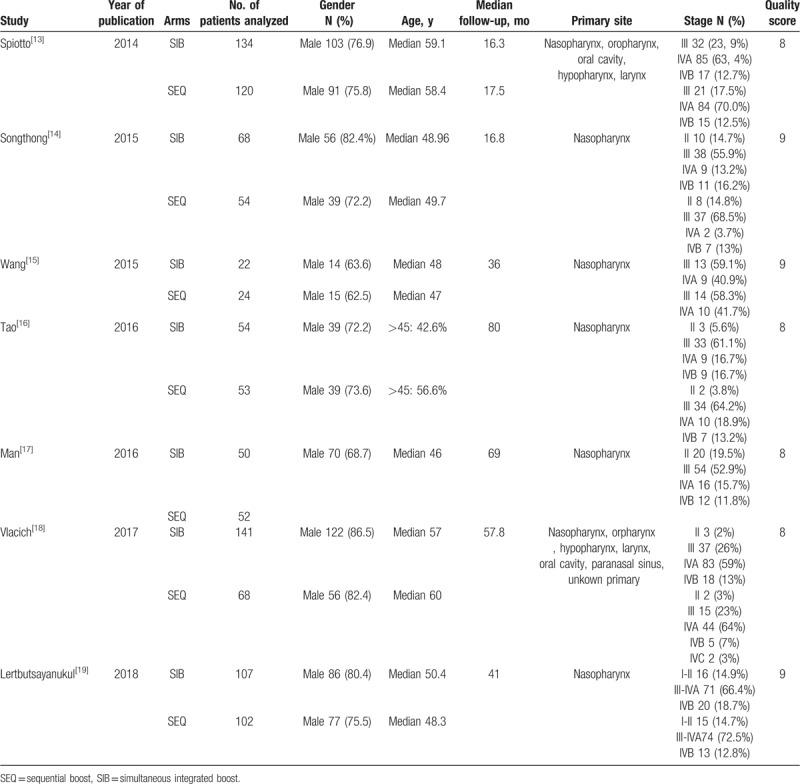
The basic characteristics of studies included.

### 3.3 Clinical outcomes and publication bias

**Figure 1:**
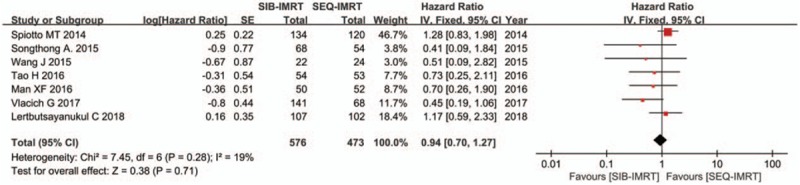
Forest plot of HR for OS.

**Figure 2:**
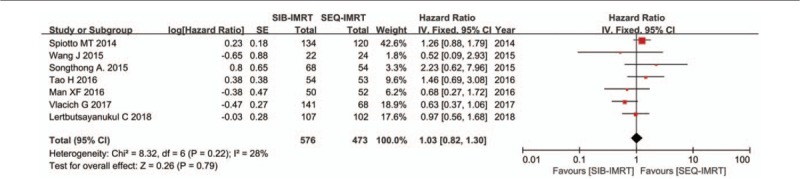
Forest plot of HR for PFS.

**Figure 3:**
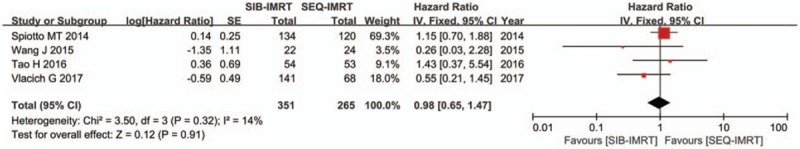
Forest plot of HR for LRFS.

**Figure 4:**
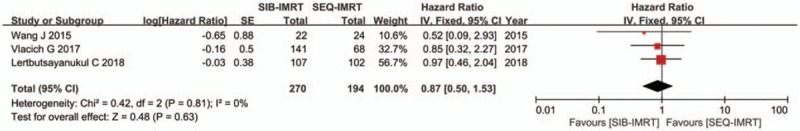
Forest plot of HR for DMFS.

**Figure 5:**
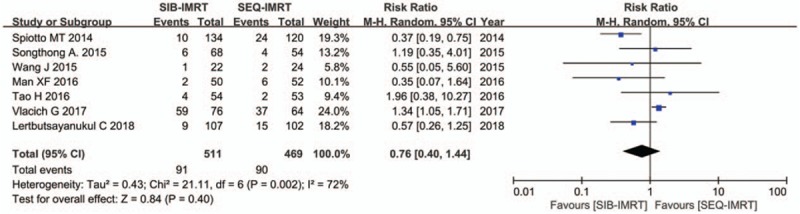
Forest plot of RR for grade ≥3 dermatitis.

**Figure 6:**
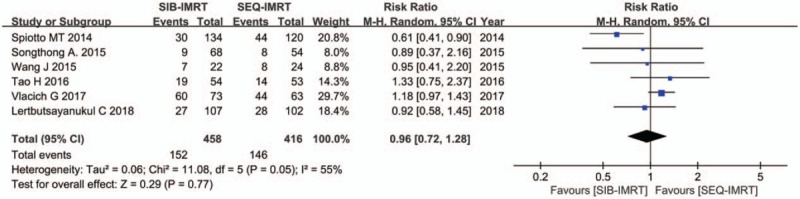
Forest plot of RR for grade ≥3 mucositis.

**Figure 7:**
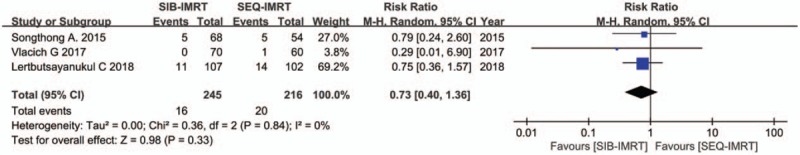
Forest plot of RR for grade ≥3 xerostomia.

**Figure 8:**
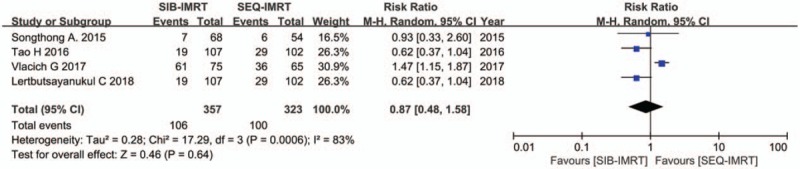
Forest plot of RR for grade ≥3 dysphagia.

The treatment data of each one of the included studies are set out in Table 2. The number of patients included to compare SIB-IMRT and SEQ-IMRT was 1049 patients for OS and PFS, 616 patients for LRFS, and 464 patients for DMFS. The natural log of the HR (lnHR) and its standard error (SE) were applied to compute the pooled HR, which evaluated the comparison between SIB-IMRT and SEQ-IMRT. The results of the pooled HR (95% CI) were: HR 0.94 (95% CI 0.70–1.27; *P* = .71) for OS, HR 1.03 (95% CI, 0.82–1.30; *P* = .79) for PFS, HR 0.98 (95% CI, 0.65–1.47; *P* = .91) for LRFS, and HR 0.87 (95% CI, 0.50–1.53; *P* = .63) for DMFS (Figs. 1–4). The risk ratio (RR) with 95% CI was calculated to compare the severe acute adverse effects (grade ≥3) of SIB-IMRT and SEQ-IMRT. As a consequence, the results of RR (95% CI) were: RR 0.76 (95% CI, 0.40–1.44; *P* = .40) for dermatitis, RR 0.96 (95% CI, 0.72–1.28; *P* = .77) for mucositis, RR 0.73 (95% CI, 0.40–1.36; *P* = .33) for xerostomia, and RR 0.87 (95% CI, 0.48–1.58; *P* = .64) for dysphagia (Figs. 5–8). The above results showed no significant difference in survival as well as in severe acute adverse effects between the SIB-IMRT and SEQ-IMRT.

**Table 2:**
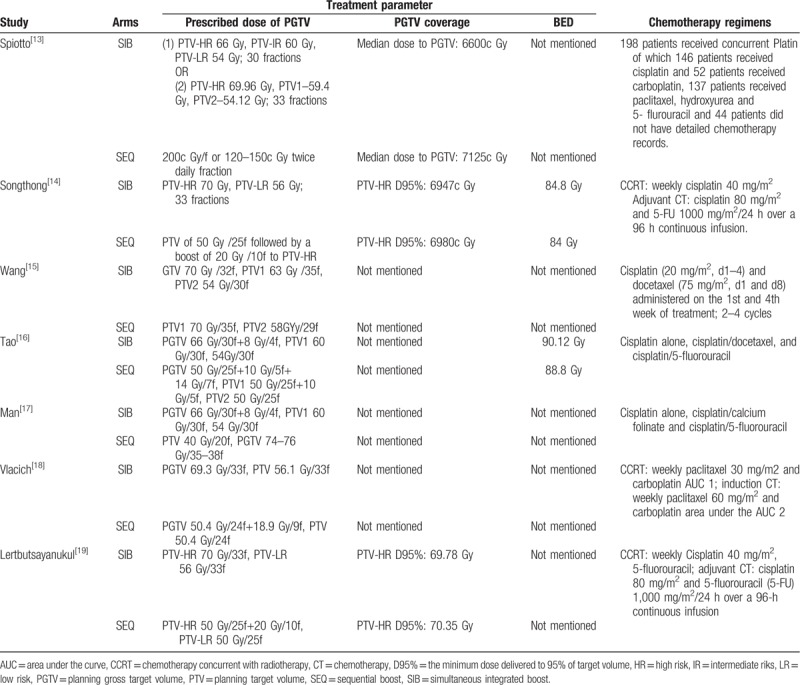
Treatment plan of IMRT and chemotherapy regimens.

In regard to the above, heterogeneity existed in the analyses of dermatitis (*I*^2^ = 72%, *P* = .002), mucositis (*I*^2^ = 55%, *P* = .05), and dysphagia (*I*^2^ = 83%, *P* = .01). Thereby, a random-effects model was utilized. Subsequently, a sensitivity analysis was used to detect the source of heterogeneity. Although, excluding one study by Spiotto et al did not change the RR of mucositis (1.13; 95% CI, 0.96–1.33; *P* = .14), the heterogeneity disappeared (*I*^2^ = 0%, *P* = .75).^[13]^ Heterogeneity for RR of dermatitis alleviated when one study by Vlacich et al was excluded (*I*^2^ = 7%, *P* = .37), whereas the RR changed significantly (0.57; 95% CI, 0.36–0.90; *P* = .02).^[18]^ Moreover, exclusion of one study by Vlacich et al lowered the heterogeneity for RR of dysphagia (*I*^2^ = 0%, *P* = .78) with RR significantly changed (0.65; 95% CI, 0.46–0.92; *P* = .01).^[18]^ It was indeed shown that there was a potential publication bias among the included studies (Supplementary Figures 1–8).

## 4 Discussion

This meta-analysis constituted a study to compare the clinical outcome of the SIB-IMRT and SEQ-IMRT based on 7 clinical researches. The present meta-analysis concluded that the survival for HNC including OS, PFS, LRFS, and DMFS was similar between the SIB-IMRT and SEQ-IMRT. In addition, it was found that both IMRT techniques tended to cause parallel frequent and serious adverse side effects.

In recent years, there have been many new advances in cancer mechanisms and treatments.^[20,21]^ It is well established that IMRT, which can not only improve local control and even patient survival but also decrease the treatment's adverse effects, remains as the optimal radiation technique for locally advanced HNC patients.^[22]^ As it is well known, 2 kinds of planning target volume (PTV) are generally applied for HNC: PGTV for boost is generated by adding a margin to the GTV and PTV by including elective volumes. The IMRT technique, which is called SEQ boost, has been applied using a shrinking field to make a sequential boost without increasing the dose to OARs, and as a result 2 to 3 treatment plans are created for each patient. The simultaneous integrated boost (SIB) allows a single plan with different doses appropriate for selective TVs; meanwhile, the normal tissues within or adjacent to the PTV-boost may receive higher doses per fraction compared to the doses delivered by SEQ-IMRT.^[23]^ On the one hand, some studies have demonstrated that SIB-IMRT could provide more conformal dose distribution, including both better coverage of boost volume and nontarget tissues sparing. A treatment comparative planning study showed that both SIB-IMRT and SEQ-IMRT provided excellent performances in terms of coverage, conformity, and dose to the PTV, whereas SIB-IMRT might be correlated with a lower rate of toxicity.^[24]^ Chen et al also reported that SIB-IMRT could allow for better distribution in the elective nodal area, whereas two-phase SEQ-IMRT could lead to higher doses to OARs regarding parotid gland and inner ear.^[25]^ An assessment of different IMRT boost techniques was implemented and the results showed that conformality and OARs sparing of the SIB-IMRT plans were superior compared with SEQ-IMRT.^[2]^ On the contrary, 2 studies indicated that the SEQ-IMRT plan, compared with the SIB-IMRT plan, not only tended to provide higher dose coverage, conformity, and homogeneity, but also significantly reduced the monitor units (MUs).^[23,26]^ Owing to the interobserver variation in the delineation of TVs and OARs by different physicians along with the different planning methods used by each physicist, dosimetric parameters of the studies were significantly different. Therefore, the quality of the treatment plan also depends on the experience and aptitude of each physician and physicist.^[27]^

Even though both IMRT techniques can reach equal dose coverage of PTV, as mentioned above, SIB-IMRT can increase the BED of delivery to the tumor with dose per fraction >2 Gy while achieving shorter treatment time. It is well established that increasing BED in HNC for local tumor control can lead to significant clinical benefits, which is associated with improved survival. When assuming that α/β is 10 Gy for HNC, it has been found that promotion of local control in HNC is approximately 1.7% per 1% change in the total dose (equivalent to 2 Gy/fraction), with that being translated to 1.2% change in BED.^[28]^ In addition, some clinical studies indicated that a notable promotion of the dose per fraction had a significantly positive impact on local control for HNC in the era of conventional radiotherapy.^[29,30]^ Nevertheless, 2 of the studies included in our meta-analysis showed that BED of SIB-IMRT was slightly higher than that of SEQ-IMRT, which could have caused a different biological effect by SIB-boost. Thus, when using SIB-IMRT, the optimum fractionation and prescribed dose are still uncertain. Furthermore, as a result of the SIB-IMRT, the PGTV can be treated with fewer fractions compared with conventional fractions (28–33 fractions compared with 35 fractions), which means less treatment time (6 weeks compared with 7 weeks).^[24]^ The rationale behind reducing the overall treatment time, in the past called as accelerated radiotherapy schedule, is based on the hypothesis that smaller treatment duration minimizes the risk of tumor regrowth during the last phase of treatment.^[31]^ Normal tissues outside the PGTV in a SIB-IMRT plan might receive low BED due to the low dose per fraction; however, normal tissues embedded in or near the PGTV might receive a higher dose.^[9]^ A dosimetry study demonstrated that SEQ-IMRT could generate better treatment plans compared with SIB-IRMT, if the PGTV was >1 cm away from at least one parotid gland.^[8]^

Growing reports have explored the optimal choice between SIB-IMRT and SEQ-IMRT, whereas the consensus remains unclear. Several studies comparing SIB-IMRT to SEQ-IMRT in HNC have been published. It was reported by most studies that SIB-IMRT could not prolong survival including OS, PFS, LRFS, and DMFS, when compared with SEQ-IMRT.^[13–19]^ Our results, which were derived of pooled HRs for survival, are consistent with the findings of previous studies. Generally, higher prescribed dose, higher dose per fraction, and shorter treatment course can increase the killing effect of radiation on tumors as well as on normal tissues. However, the clinical benefit from dose escalation may not be observed when a change in the total prescribed dose is not apparent.^[3]^ Although the prescribed dose ranging from 66 to 74 Gy delivered to GTV for HNC was widely acceptable, the prescribed dose of GTV was even lower in the SIB-IMRT plan than that in the SEQ-IMRT plan in 2 of the included studies.^[13,17]^ Eventually, this offset the advantages of the higher dose per fraction for SIB-IMRT, and might result in similar clinical outcomes between the groups. Moreover, the dose per fraction delivered to PGTV was slightly higher in the SIB-IMRT group than that in the SEQ-IMRT group which could create a survival benefit. Similarly, the difference of the overall treatment time between SIB-IMRT and SEQ-IMRT groups might not be obvious enough to cause a positive effect. As the actual dose delivered to the organs, independently of their risk state, was not calculated in the included studies, there is still confusion regarding the impact of dose to organs (such as skins) on adverse effect. Spiotto et al indicated that SIB-IMRT predicted less dermatitis compared with SEQ-IMRT.^[13]^ Conversely, 2 articles showed that SEQ-IMRT had more advantages than IMRT-SIB in protecting OARs, and that SIB-IMRT induced a higher rate of severe dermatitis and dysphagia.^[16,18]^ Stage of the disease, site of primary lesion, variation in the delineation, overall treatment time, and quality of the treatment plan might contribute to the risk of adverse effects. Thus, further studies are needed to investigate the mechanism of the adverse effect of these 2 methods. Regarding the occurrence of adverse effects, our results supported that SIB-IMRT might confer similar risk of acute severe side effect compared with SEQ-IMRT, an observation which was in line with some previous studies.^[14,16,19]^

When the dermatitis and dysphagia incidents of the SIB-IMRT and SEQ-IMRT groups were pooled, heterogeneities were noted as above. To explore the source of heterogeneity, sensitivity analyses were conducted, and showed that a particular study was responsible for the heterogeneity.^[18]^ This research analyzed HNC patients with various primary tumor sites, including nasopharynx, oropharynx, hypopharynx, oral cavity, paranasal sinus, and larynx, which contributed to the variation of the designed treatment plan. Moreover, heterogeneity of the pooled HR for mucositis was created by the study of Spiotto et al, which analyzed different number of patients with stage III to IV and various primary tumor sites.^[13]^ Besides HNC, the difference of clinical outcomes between SIB-IMRT and SEQ-IMRT has also been compared in some other malignant tumor diseases, such as breast cancer and prostate cancer. Two studies indicate that SIB-IMRT, compared with SEQ-IMRT, prominently reduced the treatment toxicity, whereas no statistically significant differences were found in survival outcomes, contrary to our findings.^[32,33]^ Therefore, SIB-IMRT may have advantages but should be applied with caution before clinical trials verify its efficacy in other tumor diseases.

The result of our meta-analysis must be viewed cautiously due to its own limitations. First, although we had tried our best to comprehensively search for studies, the study number included in the meta-analysis was small. This meta-analysis depended on the findings of randomized controlled trials together with retrospective studies which had relatively small sample sizes and suffered from confounding factors and potential bias. Second, due to objective conditions, we included patients with inconsistent clinical characteristics, such as various sites of the primary tumor. Third, variability among studies regarding radiation treatment plans, as well as different cycles and regimens of chemotherapy, might potentially stress the uncertainty of the results. Finally, publication bias, heterogeneity, and the quality of pooled studies could introduce additional limitations.

## 5 Conclusion

In conclusion, we approve the notion that SIB-IMRT provides similar treatment outcomes without compromising the risk of severe acute adverse events, compared with SEQ-IMRT in HNC patients. Both IMRT techniques tended to cause comparable frequent and serious adverse side effects. SIB-IMRT may be superior to SEQ-IMRT in its convenience and short-course of treatment, but there is still confusion in terms of optimum fractionation and prescribed dose. Based on current knowledge, SIB-IMRT and SEQ-IMRT can be suggested as a routine choice of radiotherapy for HNC. To obtain sufficient statistical power, future investigations are needed.

## Author contributions

**Conceptualization:** Rensheng Wang.

**Data curation:** Li Jiang.

**Formal analysis:** Zhendong Yang.

**Funding acquisition:** Rensheng Wang.

**Project administration:** Zhendong Yang.

**Software:** Li Jiang.

**Supervision:** Yong Zhang.

**Validation:** Yong Zhang, Zhendong Yang, Feifei Liang, Jiangtao Wu.

**Visualization:** Yong Zhang.

**Writing – original draft:** Li Jiang.

**Writing – review and editing:** Rensheng Wang.

## Supplementary Material

Supplemental Digital Content
