# Supplementary material for: A comparison of clinical outcomes between simultaneous integrated boost (SIB) versus sequential boost (SEQ) intensity modulated radiation therapy (IMRT) for head and neck cancer: A meta-analysis
Source: Medicine (Baltimore). 2019 Aug 23;98(34):e16942. doi: 10.1097/MD.0000000000016942 (PMC6716705; doi:10.1097/MD.0000000000016942)

Supplementary Figure 1 Publication bias for OS


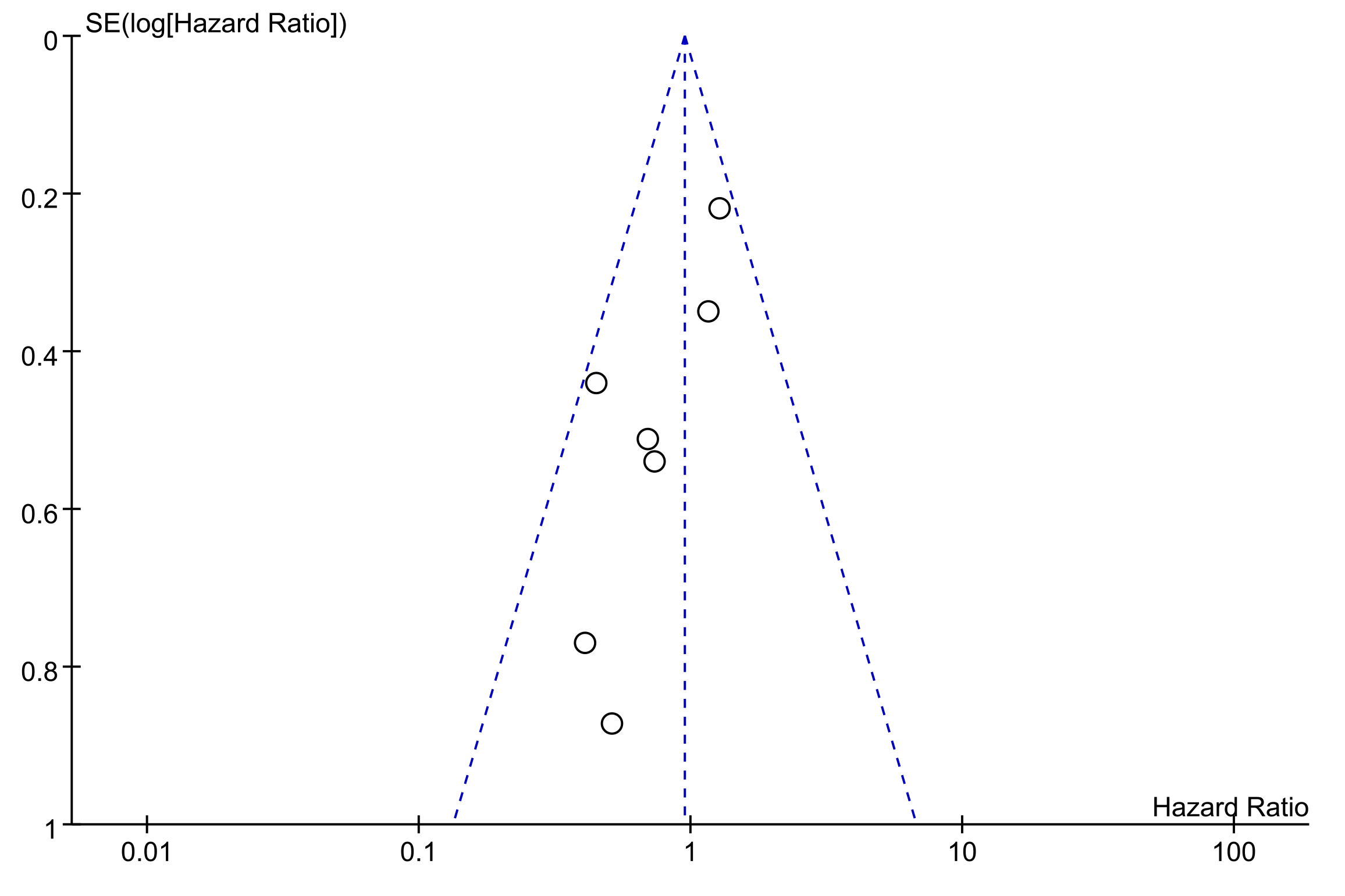


Supplementary Figure 2 Publication bias for PFS


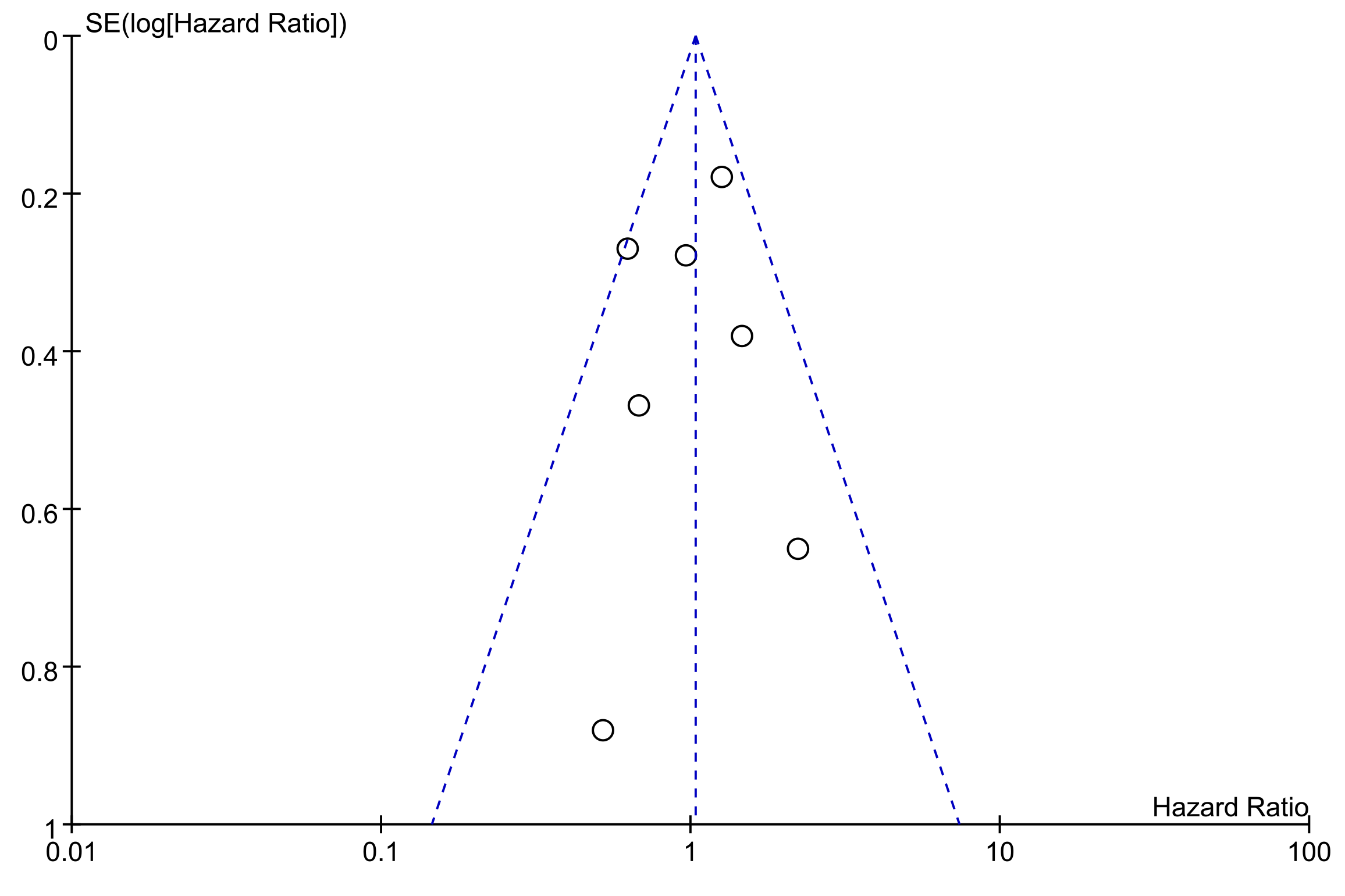


Supplementary Figure 3 Publication bias for LRFS


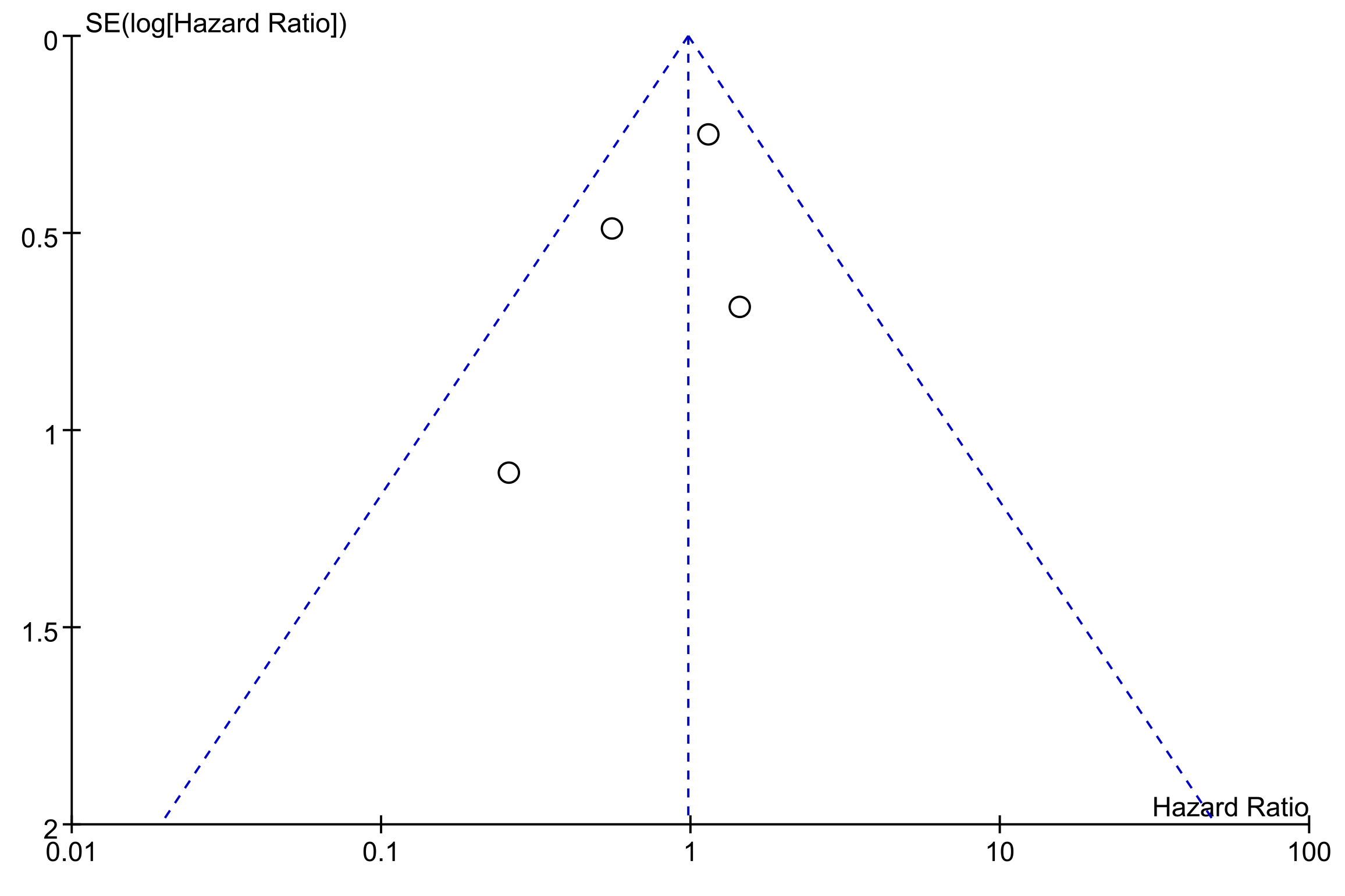


Supplementary Figure 4 Publication bias for DMFS


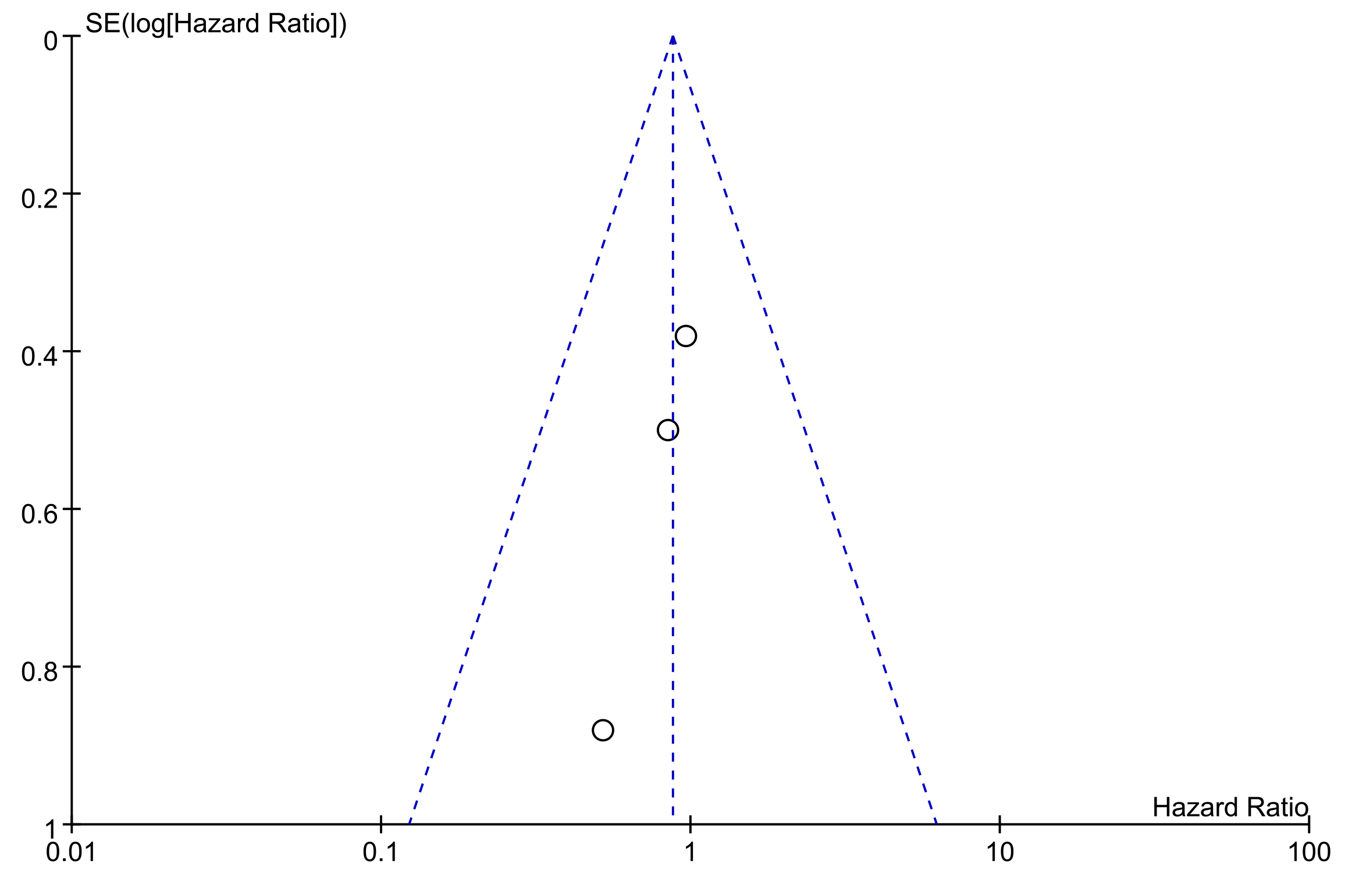


Supplementary Figure 5 Publication bias for dermatitis


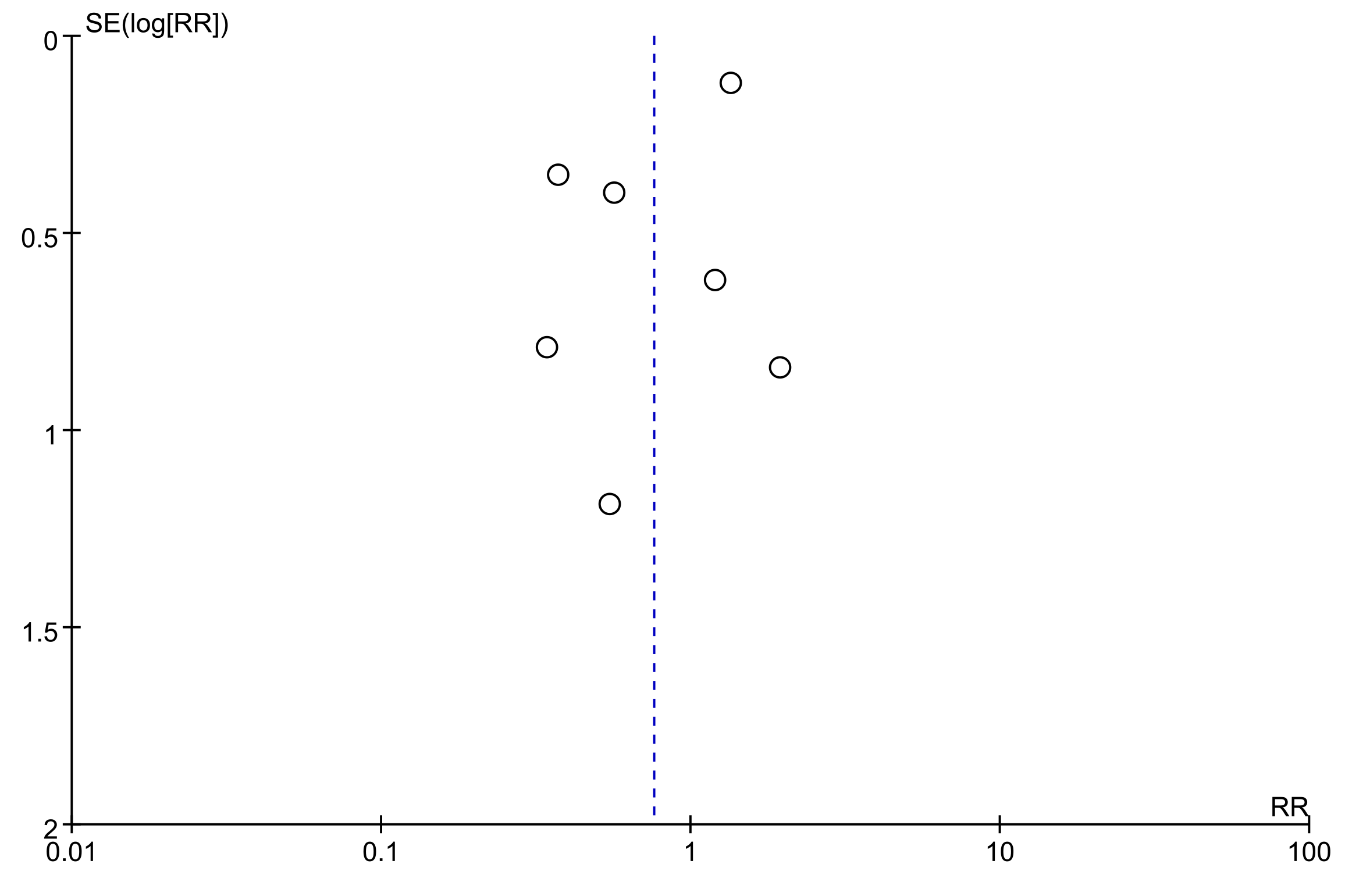


Supplementary Figure 6 Publication bias for mucositis


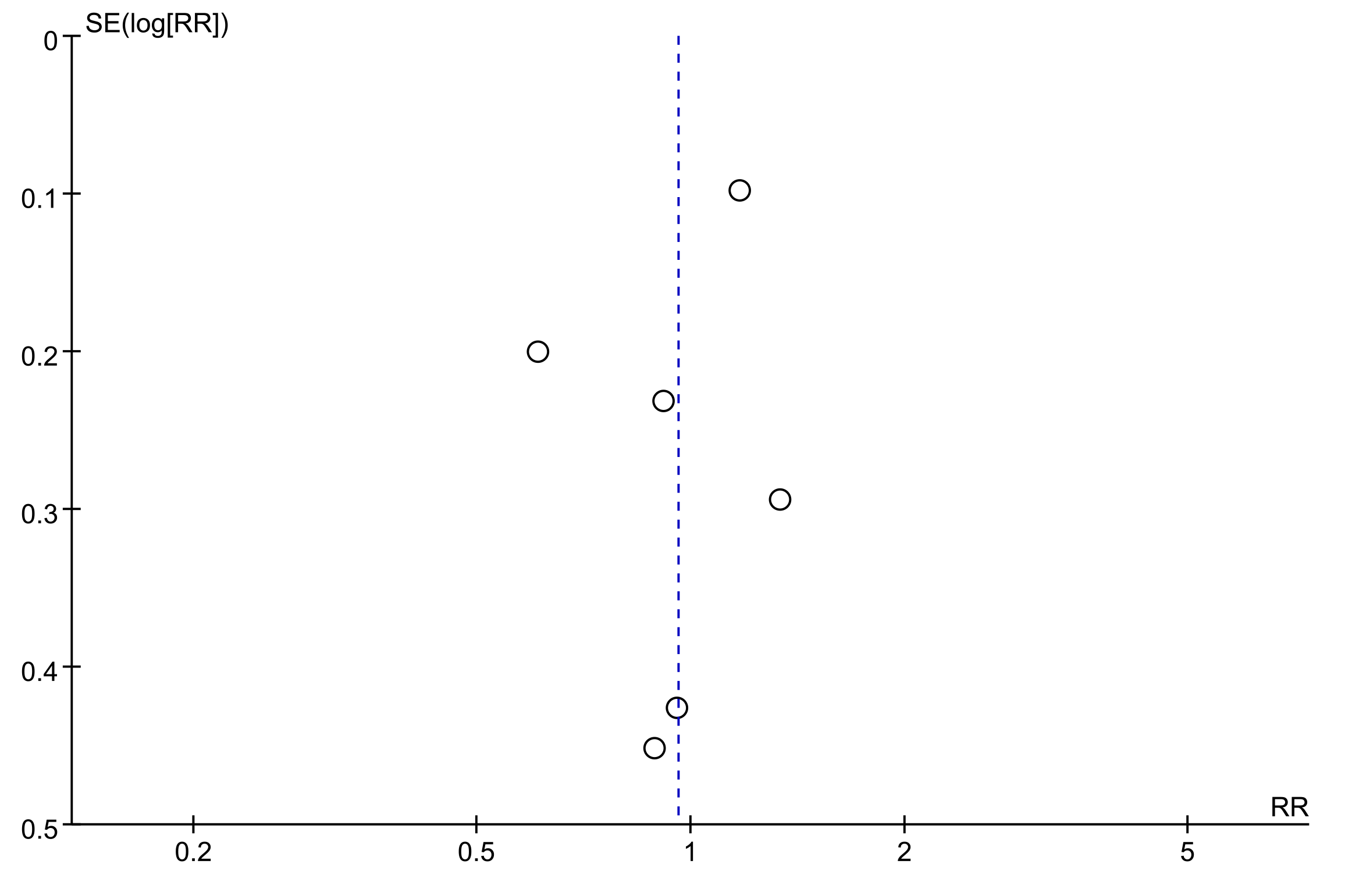


Supplementary Figure 7 Publication bias for xerostomia


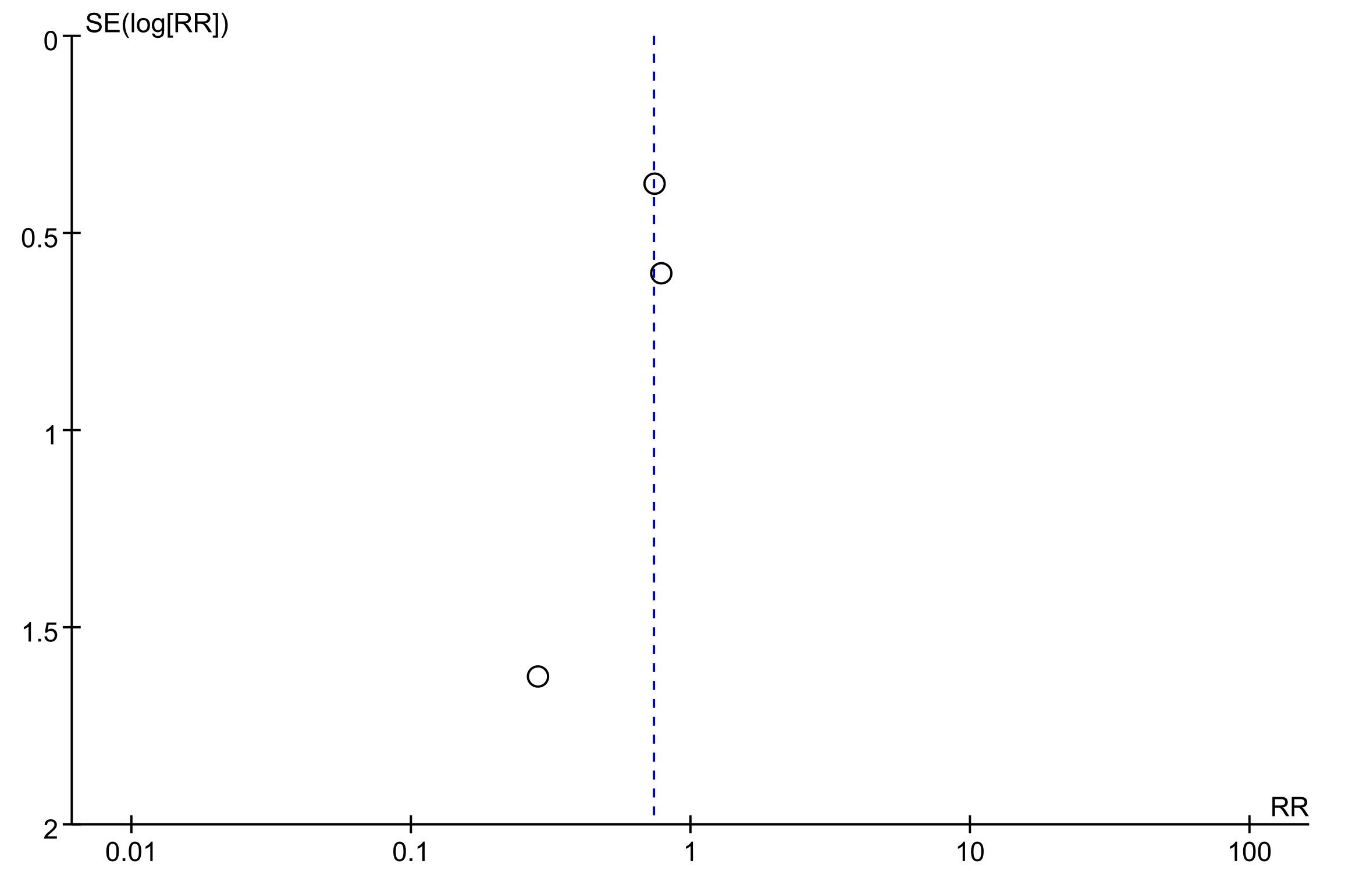


Supplementary Figure 8 Publication bias for dysphagia


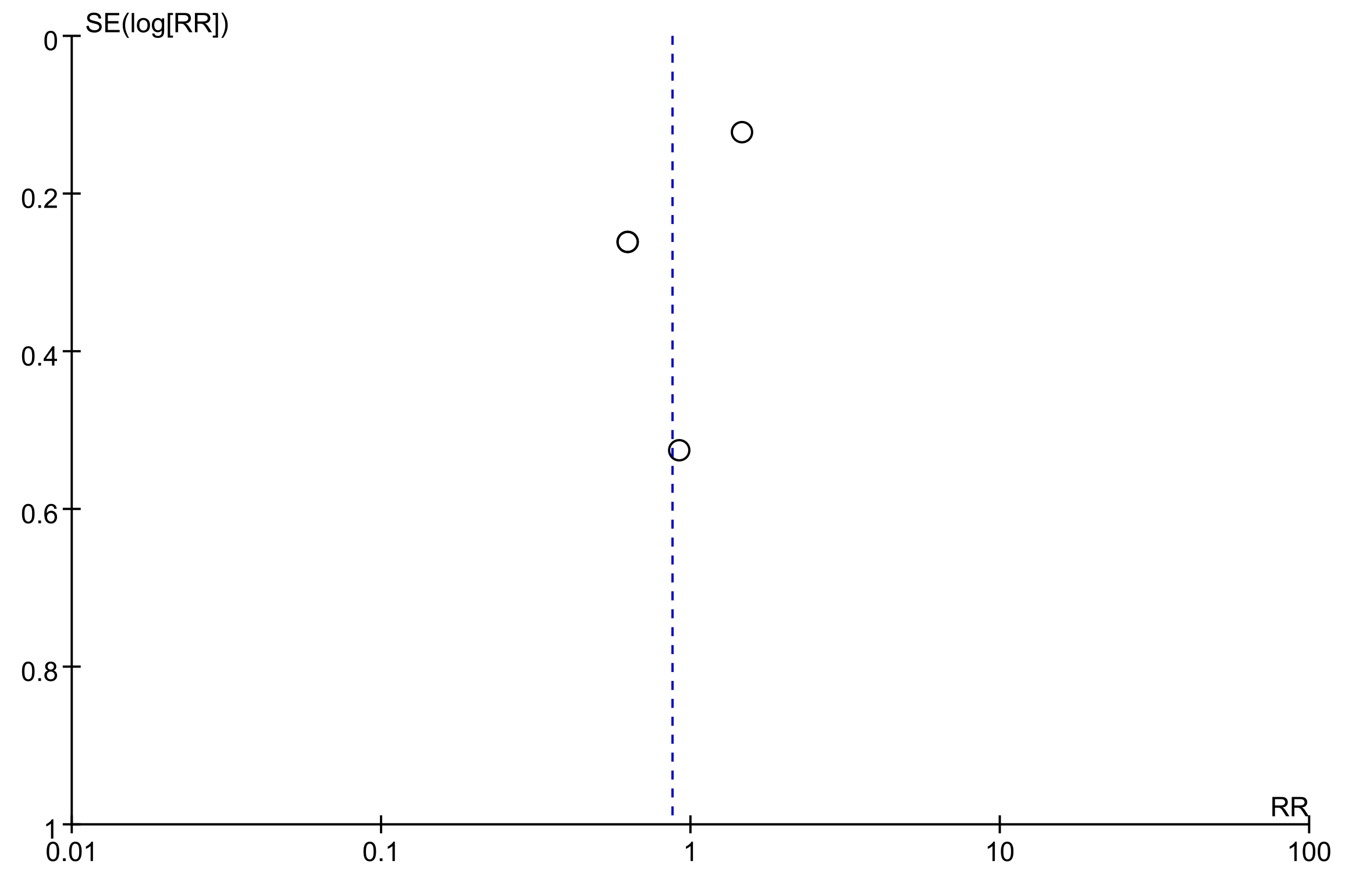

Supplement: Supplemental Digital Content [file medi-98-e16942-s001.doc]
